# Bringing Macrophages to the Frontline against Cancer: Current Immunotherapies Targeting Macrophages

**DOI:** 10.3390/cells10092364

**Published:** 2021-09-09

**Authors:** Mariana Reis-Sobreiro, Afonso Teixeira da Mota, Carolina Jardim, Karine Serre

**Affiliations:** Instituto de Medicina Molecular João Lobo Antunes, Faculdade de Medicina da Universidade de Lisboa, 1649-028 Lisboa, Portugal; mariana.sobreiro@medicina.ulisboa.pt (M.R.-S.); afonso.costa@medicina.ulisboa.pt (A.T.d.M.); cjardim@medicina.ulisboa.pt (C.J.)

**Keywords:** macrophages, myeloid-targeted therapies, reprogramming, antitumor functions

## Abstract

Macrophages are found in all tissues and display outstanding functional diversity. From embryo to birth and throughout adult life, they play critical roles in development, homeostasis, tissue repair, immunity, and, importantly, in the control of cancer growth. In this review, we will briefly detail the multi-functional, protumoral, and antitumoral roles of macrophages in the tumor microenvironment. Our objective is to focus on the ever-growing therapeutic opportunities, with promising preclinical and clinical results developed in recent years, to modulate the contribution of macrophages in oncologic diseases. While the majority of cancer immunotherapies target T cells, we believe that macrophages have a promising therapeutic potential as tumoricidal effectors and in mobilizing their surroundings towards antitumor immunity to efficiently limit cancer progression.

## 1. Introduction

Representing indispensable components of the innate immune system, macrophages possess remarkable strategic anatomical, and functional diversities [1,2], performing a plethora of activities to protect tissues when homeostasis is disrupted [3,4]. Upon an insult, macrophages rapidly evoke an immunological response to link innate and adaptive immune cells, eliminate the danger, and restore stable conditions [5,6]. Tumors are seen as a tissue with deregulated features [7] composed of tissue-resident cells and a large proportion of recruited immune cells. Strikingly, macrophages are one of the most represented immune populations in cancer tissues [8,9], and tumor-infiltrating macrophages (TAMs) thus attract a lot of attention from immuno-oncologists to understand their physiological roles in tumor biology.

Studies conducted in the 1970s and 1980s demonstrated that macrophages activated in vitro with cytokines (such as IFN-γ) and bacterial-derived products (such as lipopolysaccharide (LPS)) acquired tumor-cell killing capacities [10,11,12,13]. This initially suggested that macrophages could display tumoricidal features in the tumor bed and promote antitumor immunity. In line with this, studies in ovarian, HER2+ breast cancer, and colorectal cancer patients further revealed that macrophages associate with better therapeutic responses and increased overall survival [14,15,16]. However, the vast majority of experimental data and clinical reports indicate that, in established cancers, macrophages mainly display protumoral functions, ranging from direct interactions with tumor cells [17] to indirect shaping of a tumor-facilitating stroma [18,19]. Moreover, they can suppress local endogenous antitumor immune responses [20,21,22] and limit the efficacy of conventional and immune-modulating therapies [23,24,25]. Thus, it is not surprising that high density of macrophages in the tumor bed is associated with poor clinical outcome [14].

**Figure 1:**
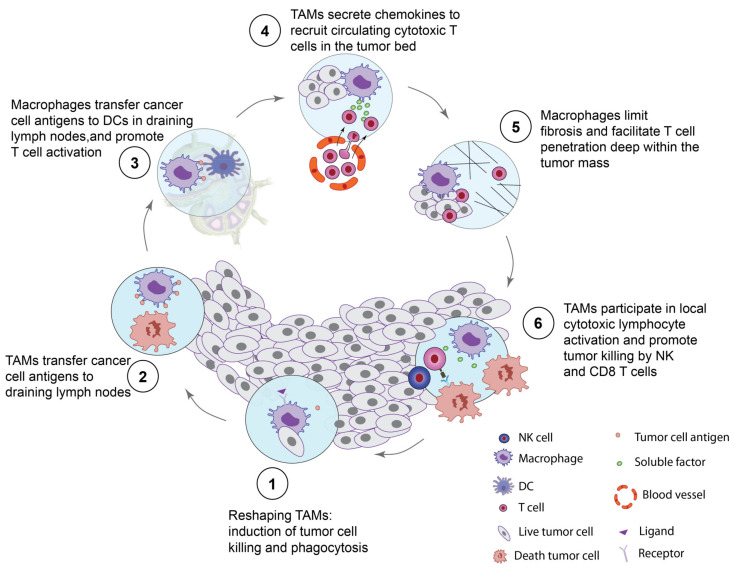
Macrophages can act as key effectors in the cancer-immunity cycle. (1) TAMs can be reprogrammed to initiate the cycle by killing tumor cells through production of ROS/NO or in a contact-dependent manner. Cytotoxic TAMs would induce the release of tumor (neo-)antigens. (2–3) Phagocytosis of dead cancer cells by TAMs and transfer of cancer-associated antigens to dendritic cells (DC), in the TME, or after migration in the draining lymph nodes (LN). Subcapsular CD169^+^ macrophages in LN have also been reported to transfer cancer-associated antigens to DC. This leads to proficient activation of tumor-specific cytolytic CD8 T cells. (4) TAMs can secrete CXCL9, CXCL10, CXCL11, the ligands of CXCR3, and actively recruit tumor-specific CD8 T cells from the circulation. (5) TAMs can produce selective enzymes that loosen up the extracellular matrix, limit fibrosis, which in turn facilitates T cell infiltration deep within the tumor mass. (6) TAMs can potentiate the cytotoxic functions of NK and CD8 T cells. Altogether, this revisited model provides the rationale for targeting macrophages, which, by boosting different steps of the cancer-immunity cycle, will accelerate the generation of an anticancer response. This figure is adapted from [26,27].

Overall, through various manipulating strategies (depleting, inhibiting recruitment or function, or re-educating), these macrophages represent attractive therapeutic targets as part of combinatorial approaches in cancer treatment. We believe that immunotherapy targeting macrophages has the potential to induce functional effectors that can actively boost every step of the cancer-immunity cycle [26,27] (Figure 1). Indeed, through their antitumor functions, macrophages have the potential to accelerate the cancer-immunity cycle to unleash a potent anticancer immune response. In this review, we provide a detailed overview of the available therapeutic strategies that target macrophages, especially their deletion, the prevention of their recruitment into the tumor, the inhibition of their immunosuppressive and tumor-promoting functions, and the reactivation of their antitumoral activities to improve current treatments.

## 2. The Multi-Functional Roles of Macrophages in the Tumor Microenvironment

### 2.1. M1/M2 Macrophage Polarizations: Classical versus Alternative

M1 (classically activated) macrophages differentiate in response to IFN-γ and LPS, whereas M2 (alternatively activated) macrophages are induced by IL-4 and IL-13. M1 macrophages express high levels of inflammatory cytokines as well as inducible nitric oxide synthase (iNOS) [28,29] and participate in the elimination of pathogens and malignant cells. M2 macrophages express a hemoglobin-haptoglobin scavenger receptor cystein-rich (CD163), the macrophage-scavenger receptor-1 (CD204), the C-type lectin mannose receptor (CD206), the macrophage receptor with collagenous structure (MARCO), arginase 1 (ARG1), and IL-10 [30], and display tissue repair and tumor-promoting features [31].

Features of the M1/M2 polarization have been associated with different cancer stages and have prognostic value. For instance, in gastric cancer patients, the median value of CD68^+^NOS2+ (M1)/CD68^+^CD163^+^ (M2) ratio was found to be a positive independent predictor of survival [32]. High M1/M2 ratios in ovarian tumor tissues are correlated with extended survival [33]. Similarly, in high-grade serous papillary ovarian cancers, a prevalence of M1 TAMs and a higher M1/M2 ratio was positively associated with longer progression-free and overall survival [16]. In addition, HLA-DR+CD68^+^ M1-like TAM levels significantly decreased during cancer progression, from pathological stage I to III in non-small-cell lung carcinoma (NSCLC) [34]. In line with this, in ovarian cancer, a high CD206^+^CD68^+^ expression is associated with high risk of disease progression [35] and high density of CD163^+^ M2-like macrophages is associated with poor prognosis [36]. In NSCLC, early in tumor formation, tissue-resident macrophages displaying the M2-like features, CD206 and MARCO, were shown to promote tumor cell epithelial–mesenchymal transition (EMT), invasiveness and to induce a Treg cell response that limits anticancer adaptive immunity [37].

### 2.2. Macrophages and Clinical Outcome

In most tumors, macrophages are one the most abundant immune cells which aid tumor development. For instance, elevated content of CD68 TAMs in biopsies is linked to unfavorable outcomes in patients with breast, bladder, gastric, pancreatic, and head and neck squamous cell carcinomas [38,39,40,41,42,43]. The detrimental prognosis in these patients can associate a high number of TAMs with various aspects of tumor progression, such as primary tumor burden, tumor-invaded nearby lymph nodes, and metastasis. This is consistent with the fact that macrophages can exhibit protumor functions, as will be detailed in Section 2.4.

However, in some circumstances, the prognostic impact of TAMs can also be associated with patient survival, such as in NSCLC, prostate, and colorectal carcinoma [14,44,45,46], and metastasis suppression in osteosarcoma [47]. TAM localization within the tumor may be an important criterion, as in lung cancer it was reported that elevated TAMs in the tumor islet (as opposed to tumor stroma) were associated with better overall survival at 3 and 5 years [48]. In gastric cancer, TAM aggregation within the cancer cell nest provided a beneficial effect in terms of tumor cell apoptosis and accumulation of CD8 T cells [49]. Thus, evidence exists that, in certain types of cancers, macrophages can display potent effector functions capable of inhibiting tumor growth, as will be detailed in Section 2.5.

Finally, macrophages can play selective roles in response to treatments. In most cases, they are reported to promote resistance to therapy. For instance, in treated mammary carcinomas, macrophages display immunosuppressive functions that hinder adaptive anticancer immunity [23,50]. Moreover, macrophages can secrete specific lipids and enzymes (such as lysophospholipids, cathepsin proteases, and cytidine deaminase) capable of interfering with chemotherapy [51,52,53]. In sharp contrast, high TAMs were independently associated with better disease-free survival in 5-fluorouracil-treated stage III colorectal cancer patients [54]. In human pancreatic ductal adenocarcinoma PDAC, high density of TAMs at the tumor–stroma interface, positively dictated prognostic responsiveness to postsurgical adjuvant chemotherapy, independently of T cell density [40]. In addition, macrophages synergize with anti-CTLA-4 immunotherapy [55] as well as actively participate in tumor cell clearance with tumor-specific mAb [56]. Altogether, these results suggest that evaluation of, not only the presence, but most importantly the phenotype, functions, and intra-tumor distribution of macrophages, in untreated and treated patients, will provide clearer prognostic and predictive values, as well as information about how to manipulate macrophages in cancer.

### 2.3. Macrophage Diversity in Cancer, Revealed by Single Cell RNA Sequencing

It became increasingly clear recently that the simple M1-M2 categorization [57] fails to portray the extent of in vivo heterogeneity of TAMs. The advent of single cell RNA sequencing (scRNAseq) applied to tumor-infiltrating immune cells has revealed that the diversity of monocyte/macrophage subsets in the tumor microenvironment (TME) does not comport with the polarization model, either as discrete states or along a spectrum of polarization trajectories in breast cancer [58].

In fact, M1- and M2-associated genes were frequently co-expressed in the same cell and positively correlated with one another along the same activation trajectory. The scRNAseq studies in various cancer types have already identified over 10 different monocyte/macrophage subsets [37,58,59,60,61]. Within colorectal cancer, small proportions of blood-derived monocytes, including CD14^+^ classical, CD14^+^CD16^+^ intermediate, and CD16^+^ nonclassical subsets were observed [60]. Further macrophage subsets were identified based on their high expression of CD68, CD163, and MRC1 (encoding CD206), which could be denoted as resident tissue macrophages and segregated into normal colon epithelial tissue (NLRP3+ and phospholipid transfer protein PLTP+) or tumor tissue (IL1B+). These subsets shared expression of proinflammatory genes such as IL1B, NLRP3, HLA-DR, but the PLTP+ subset also expressed LYVE1 and IL10, potentially holding a critical role in restraining inflammation and fibrosis. In addition, two distinct TAM subsets show inflammatory/phagocytic (C1QC^+^, TREM2, MERTK, and CD80) and pro-angiogenic/tumorigenic (SPP1+, VEGFA, and MARCO) signatures, respectively [60]. Deciphering the transcriptional trajectories of these subsets revealed that while IL1B+ TAM could give rise to C1QC+ TAMs, on the other hand NLRP3+ may give rise to SPP1+ TAMs. Anti-CD115 treatment targeting macrophages was shown to preferentially deplete the SPP1^+^ TAMs sparing the C1QC^+^ TAMs and to promote tumor regression [60]. A systematic analysis confirmed the existence of these TAM subsets across 15 human cancer types [61]. Surprisingly, the TAM subset displaying higher pro-angiogenic functions (VEGFA, SPP1, MARCO) exhibited the higher diversification of markers across different cancer types. Moreover, the composition of TAMs appeared to be associated with certain features of cancer somatic mutations and gene expressions [61]. Another study combined intracellular FACS staining against Arg1 with scRNAseq and identified a unique Trem2+CX3CR1+ TAM subset with potent immunosuppressive functions against T cells [62]. Interestingly, the authors also showed that genetic ablation of *Trem2* in mice decreased immunosuppressive TAMs and exhausted CD8 T cells, in turnlimiting tumor growth.

A novel nomenclature is starting to emerge that associates TAMs with selective gene expression profiles and effector functions. A deep understanding of the extent of the phenotypic and functional diversities of TAMs will be critical for developing effective myeloid-targeted immunotherapies.

### 2.4. Roles of Macrophages in Tumor Progression

#### 2.4.1. Tumor Angiogenesis and Local Immunosuppression

Tumor cells develop numerous strategies to grow, invade, and metastasize, in part through the secretion of chemokines. Monocytes/macrophages are actively recruited by tumor cells that produce chemokines such as CSF-1 [63], monocyte chemoattractant protein-1 (MCP-1/CCL2) [64], and CCL5 [65,66,67]. In turn, these infiltrating macrophages establish a continuous crosstalk with tumor cells producing tumor-surviving factors such as epithelial growth factor (EGF) [68,69], thymic stromal lymphopoietin (TSLP) [70], and transforming growth factor beta (TGF-β) [71] that leads to cancer cell proliferation. In line with this, we previously found that unconventional small peritoneal macrophages produce soluble unidentified protumor mediators uniquely and directly capable of promoting ovarian cancer cell proliferation [72].

Macrophages are also actively involved in remodeling the TME architecture, displaying pro-angiogenic programs to provide oxygen and nutrients to the growing neoplastic tissue [73]. Tumor angiogenesis does not result only from the interaction of cancer cells with endothelial cells, as TAMs also have a critical role in triggering the neoformation of blood vessels. They express angiogenic factors such as vascular endothelial growth factor (VEGF) [74,75], placental growth factor (PGF), and platelet-derived growth factor (PDGF) [76], in particular in response to the hypoxia present in avascular and peri-necrotic areas [77]. TIE2-expressing TAMs sense endothelial cell-produced angiopoietin 2 (ANG2), align alongside tumor vasculature, and are critical for de novo angiogenesis and cancer cell dissemination [78,79].

Macrophages are instrumental in the progression of the tumor by suppressing the antitumor action of other immune cells. This is mainly achieved by the production of various immunosuppressive cytokines. IL-10 produced by macrophages can act in an autocrine and local manner to inhibit IL-12 production [80]. The lack of IL-12 limits macrophage ability to phagocytose tumor cells and to produce pro-inflammatory nitric oxide (NO), skewing macrophages into immunosuppressive phenotypes [81,82]. On neighbor cells, IL-10 also hampers the maturation of dendritic cells (DCs) [50,83], which are indispensable for active tumor immunity. TGF-β, also produced by TAMs, prevents CD8 T cell-mediated antitumor responses [84]. Mechanisms of TGF-β immunosuppression include: inhibition of CXCR3 in CD8 T cells, thereby limiting their trafficking into tumors [85], induction of CCL22 that controls the recruitment of Treg cells to the TME [86], and promoting survival and immunosuppressive phenotype of monocytes [87].

Low availability of nutrients in the TME is an important hurdle for efficient T cell effector functions. Tumor cells and macrophages can express selective enzymes that degrade amino acids, such as indoleamine 2,3-dioxygenase (IDO) [88,89] and arginase (Arg) [90,91], which deprive the microenvironment from tryptophan and arginine, respectively, and restrain the required immunity to fight and eliminate cancer.

The immune checkpoint programmed cell death-1 (PD-1), a co-inhibitory receptor critical to maintain self-tolerance, is mainly expressed on T cells and negatively regulates peripheral T cell responses. Tumor cells frequently overexpress programmed cell death ligand 1 (PD-L1) to escape from the immune system. Importantly, TAMs are also a critical source of PD-L1 to suppress CD8 T cell function against the tumor [92]. Targeting the PD-1/PD-L1 pathway with blocking antibodies has revolutionized the treatment of a wide variety of malignancies, leading to durable therapeutic responses not typically achieved with traditional cytotoxic anticancer agents.

#### 2.4.2. Macrophages and Metastasis

At the primary cancer site, dissemination of tumor cells is often preceded by the acquisition of mesenchymal features by epithelial cancer cells, a process known as epithelial–mesenchymal transition (EMT). After intravasation into the bloodstream and extravasation to the metastatic niche, cancer cells undergo the inverted process, called mesenchymal-to-epithelial transition (MET) to establish and thrive in the new environment. Remarkably, TAMs coordinate the sequence of events throughout this metastatic dissemination process. In the primary site, TAM-secreted TGF-β can induce EMT and guide cancer cells to lose adhesion, becoming phenotypically more elongated and motile in a way that facilitates their entrance into the bloodstream [93,94]. Furthermore, TAM-derived CCL8 induces the formation of pseudopodia, which are plasma membrane protrusions, needed to increase the motility of cancer cells. Real-time imaging revealed that local and transient vascular permeability and tumor cell intravasation are stimulated by Tie2+ macrophage-derived VEGFA [95].

Pioneer work from Pollard and colleagues showed, using the PyMT transgenic mouse model susceptible to mammary cancer, that genetic reduction of macrophages drastically delayed the progression to invasive and metastatic carcinomas without affecting the incidence or the growth of primary tumors [96]. These findings led to the definition of metastasis-associated macrophages (MAMs) suited to promote breast cancer dissemination [97]. The authors further demonstrated that the MAMs expressed CD11b+F4/80+CSF1-R+CD11cdimCX3CR1highCCR2highVEGFR1high [97] as well as CD204+IL4R+ [98], and that they originated from inflammatory Ly6C+ monocytes recruited by CCL2 [98,99,100]. Mechanistically, MAMs are critical for the engraftment and growth of breast cancer clones through their capacity to produce hepatocyte growth factor [101], and to inhibit the anti-metastatic functions of NK cells via membrane-bound TGF-β expression [102]. Moreover, inflammation-induced EMT upregulates IL12Rβ2, a subunit of the IL-35 receptor, in cancer cells to help them respond to IL-35 during metastasis [103]. Then, at the metastatic site, macrophages secrete IL-35 to facilitate metastatic colonization through activation of JAK2-STAT6-GATA3 signaling, which induces MET in cancer cells.

It is estimated that 90% of cancer-related deaths are due to metastization [104]. Thus, and considering the implication of macrophages in the primary tumor as well as in metastatic dissemination, immunotherapies targeting these cells represent a critical opportunity to block the distant seeding of malignant cells and improve the survival of cancer patients.

### 2.5. Roles of Macrophages in Tumor Regression

**Figure 2:**
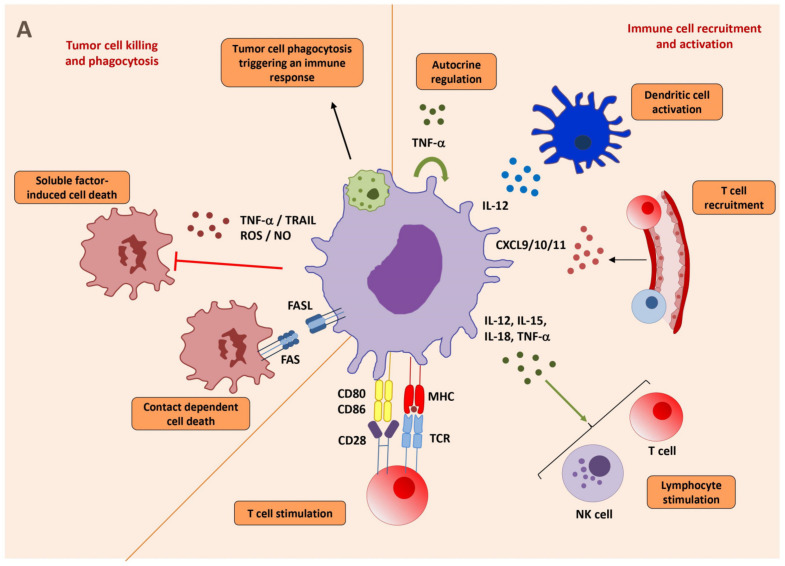

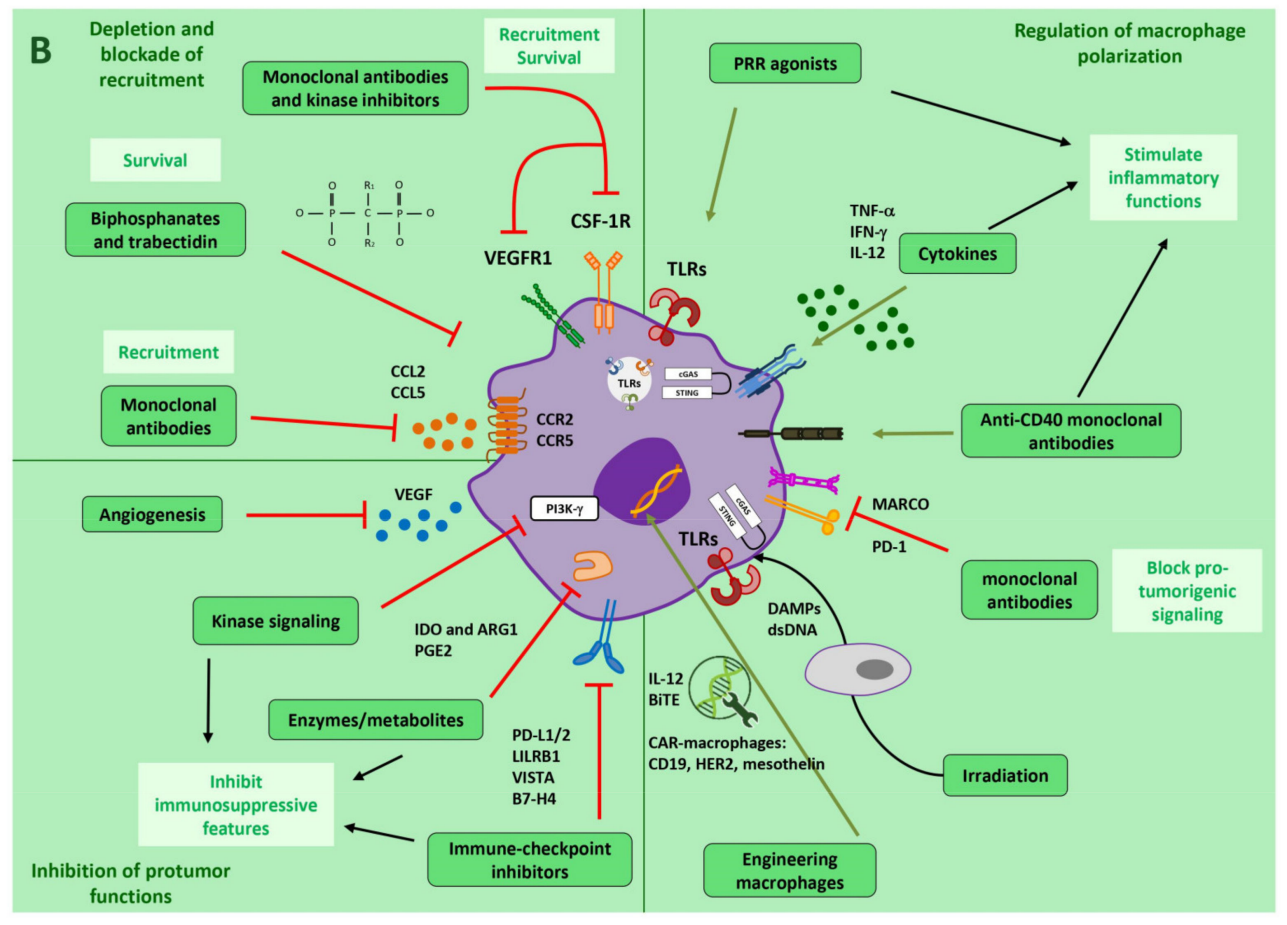
(**A**) Describes the main known antitumor functions of macrophages in particular tumor cell killing and phagocytosis as well as recruitment and activation of immune cells in the TME. (**B**) Presents the current therapeutic strategies that target TAMs to induce anticancer responses. On the top left side are different approaches to kill macrophages or inhibit their recruitment in tumors. On the bottom left side are approaches to inhibit TAM protumor functions. On the right side are strategies to re-educate TAMs into antitumor effectors.

Most of the literature has described TAMs that supports tumor progression, overshadowing that, in fact, macrophages can limit tumor growth. Some subsets of macrophages with a pro-inflammatory phenotype were observed in early stage ovarian and colorectal cancers and associated with good prognosis [105,106]. It is likely that TAMs display pro-inflammatory signatures at an early stage of tumor growth but then acquire tumor-promoting features during malignancy progression [107]. In the next sections, we aim to describe known antitumor functions of TAMs (Figure 2A).

#### 2.5.1. Properties of Macrophages to Kill and Phagocytose Tumor Cells

Macrophages can kill tumor cells through the recognition of specific cell surface markers and production of tumoricidal molecules (Figure 2A Left). They are capable of clearing apoptotic and viable tumor cells. Apoptotic cells undergo various changes such as the redistribution of phosphatidylserine and calreticulin to the plasma membrane. The recognition of phosphatidylserine for the clearance of apoptotic cells (a process termed “efferocytosis”) triggers immunosuppression and the conversion of TAMs into anti-inflammatory effectors [108,109]. In contrast, uptake through calreticulin triggers an immunogenic response against apoptotic cells [110,111]. Furthermore, apoptotic cells activate the complement and are opsonized with iC3b, leading to recognition and uptake by macrophages [112]. Macrophages can also phagocytose live cancer cells, but the latter have evolved mechanisms to escape immune phagocytic recognition and overexpress “don’t-eat-me” signals. CD47 is a marker of “self” expressed on normal cells, that, when binding to signal regulatory protein alpha (SIRPα, CD172a) on the surface of immune cells hinders phagocytosis [113]. The contribution of TAMs, in tumor cell elimination, was revealed in mouse models of tumor cells that became insensitive to cytotoxic CD8 T cells [114]. In line with this, the success of mAbs targeting tumor surface antigen, which represents a powerful strategy for the treatment of several types of cancer, relies on the Ab-dependent cellular cytotoxicity (ADCC) exerted by macrophages, which express activating FcR. An elegant study, using intravital imaging in a model of B cell lymphoma, demonstrated the key role of macrophages in tumor cell elimination in response to anti-CD20 rituximab in the bone marrow [115]. Fas ligand (FasL), a cell surface molecule belonging to the tumor necrosis factor family, binds to its receptor Fas, mediating apoptosis by caspase activation. In human colorectal cancer, macrophages were found to be the main source of FasL and they associated with apoptotic cancer cells along the invasive margin [116]. However, the expression of FasL by TAMs has been mainly associated with immunosuppressive roles. FasL expression by TAMs was found to serve as a barrier against the infiltration of CD8 T cells [117]. In patients with liver metastasis, FasL+CD11b+F4/80+ macrophages could directly eliminate Fas+CD8 T cells [118].

Pro-inflammatory macrophages acquire the capacity to express iNOS and produce the free radical NO. Diffusion of NO in their proximity result in tumor cell death [119,120]. In addition, macrophage-derived NO was found to induce adhesion molecules on tumor vessels favoring T cell extravasation and tumor rejection [121]. However, NO produced by TAMs has also been linked to resistance to therapy [122] and to suppression of T cell functions [123]. This suggests a bimodal dose-dependent effect, with NO at moderated concentration may display tumor cell killing properties, while at high concentration in the TME, NO might be propagating tumor-promoting effects. Finally, TNF-related apoptosis-inducing ligand (TRAIL) is a cytokine that can re-educate TAMs to a M1-like phenotype and induce apoptosis of tumor cells through the binding to death receptors DR4 and DR5 [124,125,126].

#### 2.5.2. Macrophages Activate CD8 T Cells and NK Cells

Macrophages are central to shaping a tumor-limiting or tumor-promoting TME due to their capacity to recruit and/or interact with various immune compartments (Figure 2A Right). For instance, TAMs produce the ligands for CXCR3 (mainly CXCL9 and CXCL10), which were found to associate with high levels of infiltrating T cells in human solid cancers [127,128]. Importantly, the expression of CXCL9 by TAMs promotes the recruitment of CXCR3-expressing CD8 T cells that are critical for the response to immune checkpoint blocker (ICB anti-PD-1/anti-CTLA-4 or anti-PDL-1) treatments [129,130].

Macrophages also produce cytokines that participate in CD8 T cell activation. Interleukin-12 (IL-12) is a key cytokine that acts on macrophages themselves, inducing a pro-inflammatory phenotype characterized by TNF-α, IL-15, and IL-18 production in the TME [131]. Furthermore, IL-12-stimulated macrophages are tumoricidal in a cell–cell contact-dependent manner, leading to a T cell-dependent eradication of established tumors [132]. Enforced activation of Notch signaling also increased IL-12-producing antitumor macrophages to ultimately limit tumor growth [133]. IL-12 produced by TAM locally enhances the inflammatory Th1 response, which in turn generates large amounts of IFN-γ and activates NK and CD8 T cells that exert antitumor effects. Then, and as detailed above, IFN-γ propagates macrophage activation. TNF-α is produced by activated antitumor macrophages. Kratochvill et al. described that the loss of the TNF-α receptor in macrophages results in the expression of genes related with protumoral functions. In this model, the polarization status of macrophages was dynamic and dependent on the balanced levels of TNF-α and IL-13 (that induces M2-like macrophages) [134]. Activated macrophages can also foster the antitumor potential of NK cells. We found that patrolling monocytes making IL-15 activate NK cells and IFN-γ production, that then inhibit lung metastases [135]. In addition, increased IL-15, IL-18, and type I IFN secretion induced NK cell-mediated cytotoxicity against tumor cells in an NKG2D-dependent manner [136,137].

Macrophages have been suggested to be as efficient as DCs at presenting tumor antigens to T cells in the TME [138], in particular after being activated with TLR agonists [139]. Interestingly, the intratumoral injection of apoptotic tumor cells with IL-2 led to an 80% rate of cure in mice models, confirming that the APC in the TME retain the intrinsic capacity to uptake, present, and generate a tumor-specific cytotoxic T cell response [140]. Macrophages are also known to interact with other immune cells in lymphoid organs. Subcapsular CD169^+^ macrophages in regional lymph nodes correlated with CD8 T cell infiltration in melanoma and breast cancer, which associated with better prognosis and improved survival rates [141,142]. These CD169^+^ macrophages were found to phagocytose dead tumor cells transported via lymphatic flow and to cross-present tumor antigens to CD8 T cells [143]. Moreover, a close collaboration between CD169^+^ macrophages and DCs was also proposed for the initiation of effective CD8 T cell responses, in which macrophages transferred Ag to DC in a cell–cell contact dependent manner [144].

Overall, macrophages are highly polyfunctional in the TME and we are convinced that this versatile multi-tasking feature is a critical property that points out macrophages as key effectors with therapeutic potentials. Multiple actions of macrophages in the TME, and in the regional lymph nodes, may positively accelerate each of the consecutive functional steps of the cancer-immunity cycle, including: (1) killing of cancer cells, (2) cancer cell antigen transfer to DCs, (3) T cell activation, (4) recruitment of circulating T cells to the tumor bed, (5) facilitating T cell infiltration within the tumor and (6) promoting the killing of tumor cells by NK and CD8 T cells (Figure 1).

## 3. Tumor Therapies Targeting Macrophages

Intense efforts have been made intending to manipulate TAMs, in particular strategies to deplete them, to limit their recruitment to the tumor site, or to exploit their plasticity to repolarize them from immune suppressive towards inflammatory and tumoricidal phenotypes. In the next sections, we aim to describe the actual therapeutic options to manipulate macrophages (see Figure 2B for a summary and Table S1 for examples of current interventional clinical trials targeting macrophages).

### 3.1. Depletion of Macrophages

#### 3.1.1. Bisphosphonates

Bisphosphonates, such as clodronate and zoledronate, which are extensively used to treat diseases associated with bone loss such as osteoporosis, are also used to deplete macrophages [145]. The administration of clodronate or zoledronate to multiple myeloma or mammary tumor-bearing mice leads to a reduction in protumoral TAMs and tumor vascularization, consequently increasing mice survival [146,147]. In F9 teratocarcinoma and A673 rhabdomyosarcoma mouse models, combination of bisphosphonates with VEGF-blocking antibodies led to TAM depletion and tumor regression, although only sustained during the time of the therapy [148].

#### 3.1.2. Blocking of Survival Signals

Cells of the monocyte-macrophage lineage rely on the macrophage-colony stimulating factor (M-CSF), also known as colony stimulating factor-1 (CSF-1), a growth factor essential for their survival and they exclusively express the CSF1R (CD115). Genetic deletion of *Csf1r* or *Csf1* results in loss of monocytes and tissue macrophages, but experiments in these mice are difficult to interpret because of additional severe pleiotropic effects, including infertility, osteoporosis, neuronal defective development, low body weight, and severe skeletal abnormalities [149,150]. The therapeutic capacity of a CSF1 signaling blockade to modulate macrophage survival was demonstrated using specific kinase inhibitors acting on CSF1R (GW2580, AMG820, and PLX3397) or blocking anti-CSF1R mAb (RG7155). Notably, administration of a blocking anti-mouse CD115 antibody to MMTV-PyMT mice delayed tumor manifestation and prolonged mice survival, simultaneously potentiating the anticancer effect of Paclitaxel [151]. Aside from breast cancer, CSF1R blockade appeared to be sufficient to enhance survival in various cancer mouse models [152,153,154,155]. In a mouse model of colon cancer, treatment with RG7155 resulted in strong reduction in TAMs accompanied by an increase in T cell infiltration [154]. Moreover, GW2580 reversed the resistance of pancreatic tumor cells to conventional chemotherapy [156]. Combination of CSFR1 inhibitor (PLX3397 or anti–CSF1R Ab) with anti-PD1 reduced TAM numbers, enhanced CD8 T cell infiltration, and consequently decreased tumor size [157,158]. In human, CSF1R inhibitors (AMG820 and PLX3397) induced a decreased in CD14^dim^CD16^+^ monocytes [159,160,161], but this effect was accompanied by an increase in plasma CSF1 and this questions the impact of treatment discontinuation on monocyte number and phenotype.

### 3.2. Blocking Recruitment to the Tumor

#### 3.2.1. CCL2/CCR2

Monocytic cells express the chemokine receptor CCR2 that confers chemotaxis to CCL2, a well-studied chemokine that regulates migration to peripheral inflamed tissues. In mice, the deletion of *Ccl2* or *Ccr2* inhibited the accumulation of macrophages in the nitrosamine-induced esophageal tumors, which potentiated the antitumor efficacy of CD8 T cells [162]. Systemic delivery of blocking anti-CCL2 antibody with docetaxel significantly reduced prostate cancer burden and prolonged mice survival, compared to single agent treatments [163]. Inhibition of CCL2 led to a diminution in metastatic seeding, which in some circumstances associated with a decrease in the primary tumor growth [99,164,165], although not necessarily [166]. Blocking of CCL2 may control TAM accumulation in primary tumors, impacting blood vessel leakiness and circulating tumor cells, without directly preventing the primary tumor growth.

However, there is a critical drawback to the usage of CCL2/CCR2 blockade, as cessation of the treatment was shown to accelerate lung and liver metastasis and precipitate death [166]. Phase I and II clinical trials with anti-CCL2 mAb failed to show significant antitumoral activity but demonstrated that the effect of CCL2 suppression is transient and associated with a rapid rebound of circulating monocytes [167,168,169].

#### 3.2.2. CCL5/CCR5

CCL5 (RANTES), which is recognized by CCR1, CCR3, and CCR5, plays important roles in the recruitment of monocytes to tumor sites. Breast cancer cells that secrete CCL5 promote macrophage ability to help local invasion and metastization [170]. Triple-negative breast cancer (TNBC) 4T1 cell line grew less in *Ccl5*-null animals because of increased tumor-infiltrating cytotoxic CD8 T cells and decreased Treg cells in tumor-draining lymph nodes [171]. Targeting the host CCL5, via nanoparticle-delivered siRNA, in combination with Maraviroc (an FDA-approved CCR5 inhibitor) resulted in reductions of circulating immunosuppressive monocyte and neutrophils in both 4T1 and PyMT tumor-bearing mice, enhancing robust antitumor responses [171]. In a murine PDX model of human malignant phyllodes tumor, blocking the CCL5–CCR5 axis by Maraviroc prevented recruitment of monocytes to the tumor and dramatically suppressed tumor growth [172]. Mechanistically, CCL5 bound to CCR5 on macrophages and activated AKT signaling to recruit and repolarize TAMs. Then, macrophages released CCL18 that enhanced and maintained cancer cell differentiation and invasion. Furthermore, CCR5 blockade induced a phenotypic shift of TAMs towards an antitumor profile via STAT3/SOCS3 transcriptional activity [173].

### 3.3. Inhibition of Macrophage Tumor-Promoting Functions

#### 3.3.1. Blocking PD-1/PD-L1 Signaling

The expression of PD-L1 is low on bone marrow monocyte/macrophage progenitors but is increased on circulating monocytes of cancer patients and is high on TAMs [174,175]. The treatment of tumor-bearing mouse and cancer patients with anti-PD-L1 mAb limited TAM-induced immunosuppression and increased survival [176]. Furthermore, blocking anti-PD-L1 antibody shifted TAMs towards a pro-inflammatory phenotype in an IFN-γ dependent manner [177].

#### 3.3.2. Kinase Signaling Inhibitors

Receptor tyrosine kinases are important for monocyte recruitment into the tumor and for the induction of protumoral functions in TAMs. Examples of tyrosine kinase inhibitors were described in Section 3.1.2 to inhibit CSF1R, but many others control protumor functionalities of TAMs.

The overactivation of the EGFR-signaling pathway is frequently involved in tumor initiation, growth, and metastasis. An anti-EGFR mAb (cetuximab) is used in patients with colorectal or head and neck cancers to inhibit tumor cell proliferation. However, EGFR is also expressed on TAMs [178], and cetuximab is also likely to act directly on EGFR+ TAMs by inhibiting the EGFR/IL-6 axis in protumor subsets and induce antitumor effectors [179,180]. In addition, cetuximab is an IgG1 mAb, an isotype that has high affinity for all FcγRs and is a potent activator of ADCC. Therefore, another layer of action is that cetuximab-opsonization of tumor cells may favor antitumor ADCC by FcγR+ macrophages and/or NK cells. This is consistent with studies demonstrating that both ADCC and cetuximab-induced macrophage responses were more pronounced in carriers of FcγRIIIa 158-Val (a polymorphic variant of the FcγRIIIa) [181,182]. However, the level of cooperation between direct EGFR inhibition in tumor cells versus cetuximab-induced immune killing of tumor cells may depend on various parameters including isotype of the anti-EGFR mAb, level of EGFR expression on tumor cells, abundance and activity of TAMs in the TME and FcγRIIIa polymorphisms.

The trio Tyro3, Axl, and MerTK (called the TAM receptors; note this is different from TAMs) are a family of receptor tyrosine kinases with shared ligands Gas6 and protein S that skew macrophage polarization towards a protumor phenotype. Given the structural similarities of these receptors, inhibitors are not specific for a single one and may also affect the other two receptors. Nevertheless, a MerTK small molecule inhibitor has demonstrated anticancer activity in glioblastoma by reducing CD206^+^ macrophages in mouse tumor samples [183].

Signaling through PI3K in macrophages also promotes their protumoral functions [184,185]. The administration of a selective inhibitor of PI3Kγ reshaped the TME, promoted a macrophage polarization switch towards immunostimulatory programs that activated CD8 T cells, and mediated tumor regression, without having a direct effect on tumor cells. In mice, PI3Kγ inhibition also diminished the accumulation of TAMs in primary breast tumors and reduced cancer cell metastization [186]. Treatment with therapeutic doses of BKM120 (a selective PI3K inhibitor of p110α/β/δ/γ) in combination with anti-PD-1 resulted in consistent inhibition of mammary tumor growth compared with either agent alone [187].

#### 3.3.3. Blocking Angiogenesis

Elevated expression of VEGFA by TAMs is associated with increased blood vessel permeability and attraction of migratory tumor cells [95]. In a renal cell carcinoma mouse model, treatment with Sunitinib, an inhibitor of receptor tyrosine kinase including VEGFR, arrested tumor growth, diminished angiogenesis and reduced both TAMs and Treg cells in the tumor [188]. Similar diminution of immature immunosuppressive myeloid cells and Treg cells was observed in renal cell carcinoma patients [189].

Treatment with bevacizumab, a blocking anti-VEGFA mAb, of xenograft tumors of human anaplastic thyroid KAT-4 carcinoma cells reduced macrophage density and MHC-II molecules and IL-1β expressions [190]. Bevacizumab also inhibited the growth of established orthotopic MDA-MB-231 breast tumors in severe combined immunodeficiency (SCID) mice, reduced tumor microvessel density and limited the infiltration of TAMs [191]. Furthermore, blocking PGF, a homolog of VEGF, inhibited macrophage recruitment to the primary site, consequently limiting tumor growth and metastasis formation of various tumors, including those resistant to anti-VEGFR inhibitors [192]. This suggested that VEGF and PGF, despite homolog, can play non-redundant roles in the context of cancer.

Although these medications have proven to be effective for late-stage and metastatic cancers, they have been shown to cause side effects such as hypertension, artery clots, and complications in wound healing. In addition, refractoriness and therapy resistance to VEGF blockade is associated with angiogenesis reactivation, revascularization, and infiltration of myeloid cells into the TME [193,194,195]. This resistance depends on macrophage recruitment and can be overcome by macrophage depletion [196].

#### 3.3.4. “Don’t-Eat-Me” Signals: CD47/SIRPα, MHC-I/LILRB1/2, CD24/Siglec10

Cancer cells evade macrophage phagocytic activity by overexpressing CD47 [113] Thus, blocking the CD47-SIRPα innate immune checkpoint can be therapeutically exploited to potentiate the killing property of myeloid cells towards cancer cells [197,198]. This macrophage-dependent tumor destruction was shown to reduce myeloid-driven immune suppression and to stimulate type-I and type-II interferon responses, in turn stimulating adaptive T cell-mediated anticancer immunity [199,200]. Immunotherapies blocking the CD47-SIRPα pathway can be achieved with antagonist molecules binding to SIRPα on macrophages or to CD47 on tumor cells. An anti-human SIRPα mAb was shown to be inert as single agent but in combination with tumor-opsonizing Ab, augmented neutrophil and macrophage antitumor activities in vitro and in vivo [201]. Examples of antagonist anti-SIRPα mAbs currently in clinical trials are listed in Table S1. While therapies targeting SIRPα do not cause direct cytotoxicity on tumor cells, CD47-targeting therapies may induce phagocytosis and direct cytotoxicity of CD47^+^ cancer cells [198] by engaging activating Fcγ receptors on macrophages and NK cells. Hu5F9-G4 (5F9), a humanized IgG4 monoclonal antibody with high affinity for human CD47, was well tolerated and demonstrated partial remissions in ovarian/fallopian tube cancers [202]. Most importantly, anti-CD47 mAb also synergized with rituximab to eliminate B-cell non-Hodgkin’s lymphoma cells [203,204], with trastuzumab in breast cancer [202] as well as with immune checkpoint blockers in preclinical lung models [205]. Unlike SIRPα, whose expression is mainly restricted to myeloid cells and neurons, CD47 is ubiquitously expressed in many tissues (including red blood cells and platelets), and the diverse CD47-targeted therapies result in differential safety and efficacy profiles. Targeting CD47 was shown to lead to anemia and thrombocytopenia in animal studies and phase I trials. In this context, a promising recombinant SIRPα molecule fused to the human IgG1 Fc domain (TTI-621) minimally bound to human erythrocytes, thus displaying reduced toxicity in pre-clinical models, and yet efficiently bound to tumor cells [206]. The development of antagonist anti-CD47 mAbs sparing erythrocytes and platelets are being explored.

Other examples of myeloid cell checkpoints are the leukocyte immunoglobulin-like receptor B 1/2 (LILRB1/2), which are receptors expressed by myeloid cells that recognize MHC-I molecules. Disruption of LILRB1 engagement with MHC-I improved macrophage phagocytosis of tumor cells in a syngeneic, immunocompetent B16-F10 melanoma mouse model [207]. And inhibition of LILRB2 polarized TAMs towards an inflammatory phenotype, at the same time suppressing granulocytic immature immunosuppressive myeloid cell and Treg cell infiltration into tumors [208]. Furthermore, the inhibitory receptor sialic-acid-binding Ig-like lectin 10 (Siglec-10), which is expressed by TAMs, can promote phagocytic evasion when engaged with CD24 expressed on tumor cells [209]. Genetic ablation and therapeutic blockade of CD24 resulted in a macrophage-dependent reduction of breast MCF-7 tumor growth in vivo and increased survival in xenograft mouse models.

Moreover, if efferocytosis triggers immunosuppression and conversion of TAMs into an anti-inflammatory subset, uncleared apoptotic tumor cells undergo necrosis and release damage-associated molecular patterns (DAMPs) and double-stranded DNA (dsDNA) that activate the innate immune system and induce adaptive antitumor activity [210]. Therefore, blockade of efferocytosis in the tumor tissue using anti-phosphatidylserine strategies could switch the immunogenically silent cell apoptosis to an immunogenic cell death, leading to robust innate and adaptive antitumor immunity [210,211,212].

#### 3.3.5. Prostaglandin E2 Inhibitors

Cyclooxygenase-2 (COX-2) is an important enzyme of the biosynthetic pathway of prostaglandins, which are known regulators of inflammation in many disease contexts including cancer. The overexpression of COX2 in TAMs is correlated with poor prognosis in breast cancer patients [213]. COX-2 activity in melanoma cells, through production of prostaglandin E2 (PGE2), promoted macrophages that displayed tumor-promoting inflammation [214,215]. TAMs that express PGE2 receptors were immunosuppressive, upregulated PD-L1, and associated with tumor progression [216,217,218]. Consequently, etodolac or SC58236, two COX-2 inhibitors, decreased protumor macrophage-related gene expression (*Ym1*, *Tgfβ*) and promoted antitumor features such as enhanced surface MHC-II IA/IE and CD80/CD86 expression and TNF-α production. Several key metastasis-related mediators, such as VEGF-A, VEGF-C, and MMP-9, were in turn inhibited, which significantly reduced lung metastasis in the 4T1 mouse model [217,219]. Another study demonstrated that the selective COX-2 inhibitor celecoxib changed the TAM phenotype from protumor to antitumor, which reduced the numbers of polyps in Apc (Min/+) mice (mouse strain with a point mutation in the *Apc* (adenomatous polyposis coli) gene that is a model for human familial multiple intestinal neoplasia (Min), adenomatous polyposis) [220]. Similarly, blocking microsomal prostaglandin E synthase-1 (mPGES-1) activity with compound III (a benzoimidazole) impaired angiogenesis, inhibited cancer-associated fibroblast accumulation, reduced tumor cell proliferation and favored a shift in the antitumor/protumor macrophage ratio, consequently reducing tumor growth [221]. Finally, diclofenac, another COX-2 inhibitor, decreased tumor vascularization by downregulating VEGF abundance in the TME, leading to a reduction of 60% of the tumor mass in a mouse model of pancreatic cancer [222].

#### 3.3.6. Inhibition of Amino Acid Metabolizing Enzymes: Arginase and IDO

In various preclinical studies, such as with Lewis lung carcinoma and 4T1 breast cancer models, Arg1/2 inhibition with *N*-hydroxy-nor-l-Arg (Nor-NOHA) restored T cell numbers and functions and consequently limited tumor growth [223]. In a urethane-induced lung carcinogenic model, treatment with 6-Gingerol an arginase inhibitor impaired lung carcinogenesis with increased abundance in iNOS+F4/80+ macrophages and decreased in arginase+F4/80+ macrophages in the lung interstitial space. The levels of IL-12 and IFN-γ increased and the levels of IL-10 and TGF-β1 decreased in the alveolar cavity, compared to those in the control group [224]. In addition, CB-1158, a potent inhibitor of arginase, also limited T cell suppression mediated by myeloid cells and reduced tumor growth in the 4T1 breast cancer mouse model [225]. This CB-1158 is being tested as a single agent and in combination with Pembrolizumab in patients with various advanced/metastatic solid tumors (NCT02903914).

In macrophages, the presence of IDO catabolizing tryptophan was linked to inhibition of T cell proliferation [226]. Consequently, treatment with the IDO inhibitor INCB023843 in combination with irradiation decreased infiltration of immature immunosuppressive monocytes and prolonged mouse survival in Lewis lung carcinoma mouse model [227]. The combination of IDO1 inhibitor GDC-0919 with anti-PD-L1 blockade increased CD8 T cell infiltration and increased antigen presentation capacity by DCs and APCs in the combination arm [228]. Unfortunately, to date, the translation to the clinic was unsuccessful and clinical trials were interrupted due to lack of efficacy [229].

Overall, we believe that macrophage functional plasticity, as described in Section 2.3, creates therapeutic opportunities for pharmacological TAM reprogramming (Figure 2B), with the objective to elicit a potent and long-lasting antitumor response.

### 3.4. Regulation of Macrophage Polarization

#### 3.4.1. Pattern Recognition Receptors

Macrophages sense “danger” [230] through the expression of pattern recognition receptors (PRR) which recognize pathogen-associated molecular patterns (PAMPs) as well as endogenous DAMPs. Importantly, engagement of these PRRs participates in breaking the tolerant response imposed by cancer and, the re-activation of the innate immune system is the first step in counteracting malignant cell progression, by making a bridge toward the induction of cancer immune surveillance [231].

##### Toll-like Receptors

Macrophages express all the 11 toll-like receptors (TLR) genes identified in human, providing some potential therapeutic targets to activate antitumor effector functions. To date, FDA-approved TLR ligands include BCG, which relies on both TLR2 and TLR4 [232], monophosphoryl lipid A (MPLA), a TLR4 ligand, and imiquimod, a TLR7 agonist. This list will certainly increase in the future for signaling via TLR3, TLR7/8, and TLR9, promote Th1 responses, and various synthetic TLR ligands have been created, several of which have been shown to have antitumor effects in clinical trials in different types of cancer.

TLR3, which mediates antiviral responses by recognizing double-stranded RNA (dsRNA), represents a valuable adjuvant that induces a Th1-type of response, adapted to fight cancer. The TLR3 agonist polyinosinic-polycytidylic acid (Poly (I:C)), an artificial dsRNA analogue, enhanced macrophage tumoricidal activity [233,234] and the antigen presentation property [235]. Several preclinical mouse models of cancer have demonstrated that TLR3 ligand induced antitumor polarization of TAMs, likely also initiating DC activation and leading to CD8 T cell stimulation [236,237]. Combination therapies are getting increased attention to overcome unresponsiveness to ICB and using Poly (I:C) with anti-PD-L1 demonstrated improved efficacy compared to the single agents alone, in three cancer mouse models (melanoma, lung, and colon) [238]. Efficient antineoplastic effects through a potent CD8 T cell response is also achieved by associating Poly (I:C) with agonist anti-CD40 or CD137 mAb [239,240]. In addition, a lot of efforts aim to improve the delivery of Poly (I:C) to malignant tissues and association with nanoplexed formulations, such as with polyethylenimine [240], assembled with poly(l-valine) hydrogel [241] or iron oxide nanoparticle [242], is an active area of investigation. Novel approaches are being tested and the inhibition of proprotein convertases (which generate substrates involved in tumorigenesis and immunosuppression) was shown to synergize with Poly (I:C) to switch macrophage phenotype and reactivate their antitumoral functions, leading to an innovative glioma therapy [243]. Many synthetic TLR3 agonists have been produced and their potential is being tested as adjuvants for vaccines or in combination with ICB, costimulatory agonist (anti-CD40), anti-angiogenic drug (bevacizumab), radiation, tumor-targeted therapy (oregovomab) or lenalidomide. One of the main strategies for current clinical trials is the administration of autologous or personalized vaccines adjuvanted with Poly (I.C), in particular in combination with ICB or chemotherapy, in cancer patients with hematologic and many types of solid cancer (Table S1).

TLR4 excessive engagement can have deleterious consequences [244], and this led to the development of TLR4 ligands with high immunogenicity and reduced toxicity. The monophosphoryl lipid A (MPLA) has been FDA-approved since 2009 as a component of the vaccine against human papillomavirus (Cervarix), a viral cause of cervical cancer [245]. Other approaches experimented in mouse models to maintain efficiency with increased tolerability are to incorporate LPS to GM-CSF-secreting whole tumor cell vector or to polymeric matrix nanoparticles, which showed increased induction of antitumor immune responses [246,247]. Current clinical trials in patients with advanced solid tumors are testing TLR4 agonists in association with agonists anti-OX40 and anti-ICOS mAbs plus Pembrolizumab (NCT03447314).

TLR7/8 agonists such as resiquimod (also known as R848; 3M-052) are potent activators of macrophages. Intratumoral administration of 3M-052 generated systemic anti-melanoma immunity and suppressed both injected and untreated tumor foci. Treated tumors accumulated antitumor TAMs, which killed tumor cells directly through the production of NO and were essential for the antitumor activity [248]. In addition, TLR7/8 agonists reprogramed protumoral TAMs into antitumoral effectors in mouse models of melanoma (B16) and colon cancer (MC38) [249] and TAMs become very efficient at antibody-mediated phagocytosis [250,251]. Thus, TLR7/8 agonists are considered valuable additional reagents with great potential in combinations with ICB and vaccination and are intensely being investigated.

TLR9, which recognizes unmethylated cytidine phosphate guanosine (CpG) oligodinucleotides, activates macrophages and acts in combination with agonist anti-CD40 mAb to induce tumoricidal activity [252]. Remarkably, CpG promotes the engulfment of CD47^+^ cancer cells [253]. An interesting feature was uncovered by intravenous injections of apoptotic bodies (used as a carrier) conjugated with CpG, which induced phagocytosis by and tumor-infiltration of inflammatory Ly6C+ monocytes [254]. Drug research is exploring means to deliver CpG directly to endosomal TLR9 in macrophages for maximal activation [255]. For instance, CpG-containing nanoparticle synergized with phototherapy and docetaxel to improve efficacy of anti-PD-L1 antibody by inducing and antitumor phenotype on TAMs, altogether leading to 4T1 tumor burden reduction [256]. Surprisingly, CpG associated with cationic agarose was efficiently recognized by Siglec-1 on the surface of tumor-draining lymph node sinus macrophages that, in turn, blocked lymphatic metastasis [257]. TLR9 agonist monotherapy is safe and combinations appear to be very promising, as demonstrated by the clinical trial testing the effect of TLR9 agonist, received intratumorally, with nivolumab and radiotherapy in chemotherapy-refractory metastatic pancreatic cancer patients (NCT04050085).

##### Retinoic Acid-Inducible Gene-I-like Receptors, and Stimulator of Interferon Genes

Retinoic acid-inducible gene I-like receptors (RLRs), are intracellular receptors that can sense nucleic acids derived from viruses, stimulate type I IFN production by macrophages, and induce antiviral innate immune responses. They are demonstrated to play pivotal roles in elevating cancer immune surveillance efficiency [258]. One current objective is to deliver RIG-I ligands to the intracellular compartment. Thus, 5’triphosphate dsRNA (3pdsRNA) encapsulated in lipid calcium phosphate nanoparticles, promoted antitumor over protumor TAMs, strong levels of pro-inflammatory Th1 cytokines, and increased the proportion of CD8 T cells over Treg cells, altogether leading to significant delay in pancreatic cancer growth [259]. In addition, pH-responsive, endosomolytic polymer nanoparticles containing 3pdsRNA activated bone-marrow derived macrophages inducing the production of inflammatory cytokines IFN-α, as well as *Cxcl10*, *Il6*, and *Tnfα* [260]. In vivo, this formulation induced immunogenic cell death in the CT26 mouse colon cancer model, which triggered pro-inflammatory cytokines (such as IFN-α), increasing CD8 T cell infiltration and leading to tumor growth delay.

Similarly, the cytosolic DNA receptor cyclic GMP-AMP synthase (cGAS) initiates a type I IFN response through the adaptor protein stimulator of interferon genes (STING) and interferon regulatory factor 3 (IRF3). In particular, STING signaling was shown to act as a key switch to augment macrophage antitumor polarization and decrease immunosuppression [261]. Furthermore, polyphyllin VII (a potential STING agonist) exerted antitumor efficacy upon macrophage priming and subsequent cytotoxic T cell intratumoral infiltration in lung cancer [261]. In addition, the blockade of efferocytosis induced STING activation and the production of type I IFN by macrophages, which in turn limited the growth of the MC38 colon mouse carcinoma, particularly in association with anti-PD-1 mAb [210].

Overall, RLRs and STING-mediated innate immune pathways have a huge potential to be part of the next immunotherapeutic strategies targeting macrophages for effective cancer treatments.

#### 3.4.2. Cytokines: IFN-γ and TNF-α

Initially called “macrophage-activating factor” (MAF), IFN-γ was recognized to promote various biological activities of macrophages such as enhanced expression of MHC-II molecules [262], release of oxygen metabolites [263] and enhanced tumor cell killing [263,264]. We have previously described the major roles of IFN-γ as a pleiotropic molecule with anti-proliferative, pro-apoptotic, and antitumor features promoting tumor immunosurveillance mechanisms [265]. Overall, despite IFN-γ’s important adjuvant potential, not only through macrophage activation but also through NK, CD4, and CD8 T cell stimulation, the translation into therapeutic application has been limited by severe toxicity after systemic administration. Various strategies aim at providing IFN-γ directly to TAMs to promote activation and antitumor functions, such as IFN-γ delivering nanoparticles [266,267]. Specifically engineered particles, referred to as a “backpack”, were also shown to evade phagocytosis and release cytokines to continuously guide the polarization of macrophages toward antitumor phenotypes in situ [268].

TNF-α is another pleiotropic cytokine that induces the activation of macrophages to a tumoricidal state [269]. Interestingly, this cytokine is also predominantly produced by macrophages, although T and NK cells are also large sources. Despite initial studies of TNF-α treatment for cancer demonstrated benefits in a significant percentage of patients with soft tissue sarcoma [270] or unresectable liver metastases from colorectal cancer [271], the held promises failed to concretize as clinical therapeutics. As for IFN-γ, a limitation of recombinant human TNF-α is high toxicity after systemic administration. Various approaches targeting the cytokine to the tumor, such as colloidal gold-bound TNF-α [272], gene transfer [273], recombinant TNF-α fused to an antibody anti-fibronectin of tumor endothelium (L19-TNF) [274], failed to reach significant results in clinical trials. Nevertheless, progress may come from oncolytic adenovirus engineered to produce TNF-α (TILT-123) [275,276] (NCT04217473).

#### 3.4.3. Antibodies: Anti-CD40, Anti-CSF1R, Anti-PD-1, Anti-MARCO

CD40 is a co-stimulatory molecule of the TNF-receptor superfamily expressed by APCs that establishes a cross-talk in which CD40-activated macrophages presenting antigen to T cells provoke upregulation of CD40L. The CD40-CD40L interaction then induces in macrophages further upregulation of MHC molecules, CD80/CD86, and pro-inflammatory cytokines, such as IL-12. These signals prime naive CD4 T cells into Th1 T cells and CD8 T cells into cytotoxic cells, the immune response favorable for tumor clearance.

Stimulation of macrophages with engineered CD40L-expressing murine lung cancer cells (3LLSA) enhanced their cytotoxic effect [277]. Then, agonist anti-CD40 mAb was shown to stimulate the tumor killing activity of macrophages [278] and to induce T cell-independent antitumor effects that involve macrophages, in neuroblastoma [279]. The tumoricidal effect was proposed to involve the production of IFN-γ, TNF-α, or NO [279,280]. Interestingly, CD40 ligation leads to a positive feedback by inducing the upregulation of intracellular TLRs, resulting in synergistic activation of both anti-CD40 and TLR (3, 7, 9) ligands in macrophages in several tumor mouse models [280,281]. CD40 agonist was also shown to synergize with chemotherapy to induce tumor regression in a genetically engineered mouse model of pancreatic cancer [282]. Engagement of CD40 permitted to overcome resistance to anti-PD1 therapy through repolarization of macrophages towards an inflammatory phenotype, leading to strong CD8 T cell activation in a intrahepatic cholangiocarcinoma [283] genetic mouse model of bladder [284] and pancreatic cancer [285]. In breast and metastatic pancreatic cancer mouse models, only combining a T cell-inducing vaccine with both PD-1 antagonist and CD40 agonist Abs was able to eradicate the majority of tumors [286]. Logically, these observations have opened the way for the development of clinically relevant anti-CD40 Abs (Table S1).

As presented above, blocking CSF1-R depletes macrophages. Surprisingly, macrophage disappearance is not immediate and CSF-1R inhibition (with BLZ945 or blocking Abs) induces a short-term rewiring of TAM functionality that promotes their antitumor functions in the glioma microenvironment [153]. Mechanistically, aside from survival, CSF1 promotes macrophage polarization toward a protumoral state [287] whereas, in turn, CSF1 withdrawal unleashes an antitumor potential before their death. This result has led to the idea of combining a proinflammatory stimulus, like a CD40 agonist, with CSF-1R blockade. This dual macrophage-targeting combination promoted antitumor TAMs and reinvigorated an effective T cell response by increasing the production of IFN-γ and TNF-α [288,289,290]. The two humanized mAb directed against CSF-1R, emactuzumab and AMG820, showed an acceptable safety profile but, unfortunately, only reached limited efficacy [160], either alone or in association with selicrelumab (anti-CD40) [291], pembrolizumab [292] or paclitaxel [293], in patients with advanced/metastatic solid tumors.

Aside from unleashing cytotoxic T cell responses, another consequence of anti-PD-1 therapy is the redirection of macrophages from protumoral to antitumoral phenotype, inducing the regression of lung metastases [294]. Surprise came, however, when PD-1 was found expressed by macrophages and to directly regulate macrophage phagocytic activities [22], as well as T cell-directed immunosuppression [295], altogether promoting antitumor immunity [296]. Even more unexpectedly, myeloid-specific PD-1 ablation was as effective as plain PD-1 knock-out (Pdcd1−/−) and was considerably more effective than T cell-specific PD-1 ablation [296]. Importantly, circulating monocytes from patients with hepatocellular carcinoma upregulated PD-1 in a severity-dependent manner [295]. Moreover, upon LPS stimulation, PD-1 positive monocytes presented lower iNOS and higher arginase 1 and IL-10 expression than PD-1 negative monocytes.

These remarkable results indicate that PD-1 plays a unique role in macrophages. Therefore, antagonist anti-PD-1 antibody may be acting not only on T cells but also on macrophages, both in the circulation and in the TME. This knowledge has already been translated into potential therapeutic approach through the development of selective macrophage-targeted PD-1 inhibition strategies. Taking advantage of the intrinsic phagocytic property of macrophages, solid lipid nanoparticle-containing PD-1 siRNA or Salmonella carrying PD-1 siRNA were capable of downregulating PD-1 expression by TAMs and limiting melanoma and colon cancer progression [297,298,299]. These proof-of-concept experiments may open up new avenues to target, at will, PD-1 inhibition in lymphocytes or myeloid cells.

MARCO is exclusively expressed by macrophages. MARCO+ TAMs display immunosuppressive features with high expression of the typical anti-inflammatory genes *arg1*, *fizz1* [300]. Consistently, MARCO expression in human solid cancers correlates with poor prognosis [20,301]. MARCO expression is induced by tumor-derived supernatant, IL-10, hypoxic conditions and IL-37 [20]. However, while a marker of immunosuppressive macrophages, MARCO engagement led to the expression of the pro-inflammatory genes *Tnf*, *Il1b*, and *Nos2*, leading to reduced primary tumor growth and metastases [300]. Interestingly, targeting MARCO by mAbs led to NK cell activation, which in turn increased their TRAIL-dependent tumor cell killing property [137].

#### 3.4.4. Irradiation

Radiotherapy that uses high doses of X-ray radiation is one of the first treatments directed to physically damage DNA and induce apoptosis of cancer cells. This cancer treatment induces an immunogenic cell death that elicits an antitumor immune response. On one hand, radiation induces the release of tumor-associated antigens into the TME as well as several endogenous TLR ligands (DAMPs); on the other hand, it induces cytosolic dsDNA accumulation that is sensed by the cGAS-STING pathway in macrophages [302]. Furthermore, radiotherapy recruits monocytes and may impact macrophage functions in situ. However, the outcome on TAMs may depend on the context and on the dose of radiotherapy. In vitro radiotherapy (2 Gy) primed macrophages towards an iNOS+ M1 phenotype [303]. Human macrophages are resistant to ionizing radiation doses (5*2 Gy) and, aside from remaining viable and metabolically active, they adopt a pro-inflammatory-like profile [304]. Furthermore, low dose of radiation (0.5 Gy) programs the differentiation of iNOS+ macrophages that orchestrate the recruitment of tumor-specific T cells, mediating tumor rejection in pancreatic carcinoma or melanoma xenotransplant mouse models [305]. In line with this, we showed that, in the 4T1 orthotopic breast tumor mouse model, radiotherapy synergizes efficiently with immunostimulatory nanoparticles to induce antitumor immunity [306].

By contrast, human irradiated (5*2 Gy) macrophages also sustain cancer cell invasion and angiogenesis [304]. This is also consistent with some preclinical models supporting a detrimental role of macrophages during radiotherapy. Glioblastoma is usually treated with conventional therapy consisting of X-ray radiotherapy associated with surgery. Irradiation (3*4 Gy) of glioma (GL261)-bearing mice led to depletion of total CD68^+^ cells but to an increase in the proportion of CD206^+^ protumor macrophages. The authors showed, using in vitro bone marrow-derived macrophages, that M0 and LPS/IFN-γ-stimulated macrophages (antitumoral) are more sensitive to X-ray radiation (2 Gy) than IL-4-stimulated macrophages (protumoral) macrophages [307]. The protumor effect of macrophages after radiotherapy was further demonstrated when, in the B16 melanoma mouse model, using clodronate-containing liposomes to deplete macrophages before radiation treatment increased the antitumor effects of ionizing radiation (20 Gy) [308]. Further work is warranted to establish the best macrophage-targeting approach, either stimulation or depletion, to synergize efficiently with radiotherapy in each type of cancer.

#### 3.4.5. Genetically Engineered Macrophages

To complement in vivo programing of macrophages, intensive research focuses on genetically engineered macrophages (GEM). This adoptive macrophage therapy would then access the tumor site or could be delivered directly within the tumor. Although this treatment may not persist for a long period of time, the anticipation is that it should allow durable local therapeutic antitumor efficacy while minimizing toxicities or unwanted on-target off-tumor effects. While genetically engineered lymphocytes, with TCR-engineered or chimeric antigen receptor (CAR) T cells, take the stage in adoptive cell therapy, genetic manipulation of myeloid cells clearly lags behind. This is due to the difficulties in expanding and maintaining macrophages in culture for genetic manipulation. However, the generation of macrophages from proliferative precursors derived from induced pluripotent stem cells [309] or monocytes [310,311,312] offer a window for transgene expression. Various approaches have been successfully used to modify macrophages, including recombinant adenoviral or retroviral vector, lentivirus-driven engineering, and CRISPR-Cas9.

Genetically engineered macrophages may be ideally suited to thrive in the TME and display antitumor functions through the secretion of pro-inflammatory cytokines or cytotoxic bispecific T cell engager (BiTE). Macrophages genetically engineered to express IL-12 reversed the immunosuppressive environment developed during metastatic progression of glioblastoma by augmenting T cell responses and reducing metastatic burden in preclinical models [311]. Macrophages engineered to secrete a BiTE specific to the mutated epidermal growth factor variant III expressed by some glioblastoma cells reduced early tumor burden in both subcutaneous and intracranial mouse models [312]. Furthermore, CRISPR-Cas9-driven epigenetic silencing of Hif1α was achieved by deletion of the histone H3 methylase EZH2, that is recruited to the Hif1α promoter region specifically. These Hif1α silenced macrophages inhibited growth of the B16-F10 melanoma syngeneic model after intratumoral injection, through reprogramming the immune suppressive TME to an active antitumoral microenvironment. This approach reduced the number of Treg cells, recruited cytotoxic T cells, and prolonged the overall survival of mice [313].

CAR-expressing macrophages may be activated in an antigen-dependent manner in the TME and display enhanced phagocytosis of tumor cells. CAR-macrophages directed to CD19 [309,310,311], HER2 [310], or mesothelin [309] to target B cell leukemia or ovarian cancer, respectively, demonstrated efficacy in mouse models. Genetic reprogramming of macrophages will undoubtedly be an important avenue of the future molecular and cellular medicine and anti-HER2 CAR-macrophages are already in clinical trials (NCT04660929).

#### 3.4.6. Intracellular Signaling, Epigenetic and Metabolic Manipulations of Macrophages

Although pharmacological inhibition of PI3K in macrophages synergized with ICB therapy to promote tumor regression [184,185], to date, there is limited therapeutic application targeting intracellular signaling (such as kinases) to reprogram TAMs. In the near future, the development of single cell proteomic profiling should permit the precise dissection of the intracellular signaling pathways that participate in the decision switch from protumor towards antitumor phenotypes. Epigenetic manipulation also represents an avenue of investigation to program macrophages and histone acetyl deacetylase (HDAC) inhibitors, which cause changes in the transcriptional profile of the macrophages, was shown to limit tumor growth [314]. Increasing interest emerges to assess the effect of diet on TAM functions. Strikingly, alternate day fasting for 2 weeks inhibited extracellular adenosine accumulation by suppressing the expression of CD73 on tumor cells, which in turn limited TAM protumoral polarization [315]. Immunometabolism is also becoming a growing area of research given that metabolism strongly connects to functionality. Metabolic reprogramming might be a proficient way to promote an antitumor phenotype in TAMs. For instance, metformin, a well-known anti-diabetic, glucose-lowering drug, induced programming toward a more antitumor phenotype of TAMs in mice, partially through AMPKα1 activation [316]. This led to the idea of combining anti-PD-1 therapy with metformin-loaded microparticles, which efficiently targeted protumoral TAMs and polarized them towards an antitumor phenotype [317]. This elegant strategy induced TME remodeling, with collagen degradation, and increased the recruitment and infiltration of CD8 T cells into tumor interiors, in a way that also enhanced penetration of anti-PD-1 antibodies.

## 4. Conclusions and Perspectives

The remarkable plasticity displayed by macrophages makes them a key nexus between the immune system and tumor cells. As such, we propose that targeting macrophages may feed the cancer-immunity cycle, initiating a snowball effect that will reshape other stromal compartments and ultimately make the TME favorable to productive antitumor actions of CD8 T cells (Figure 1).

ICB, targeting CTLA-4 or the PD-1/PD-L1 axis, has been a game changer in cancer treatments; unfortunately, this therapy prolonged the life of only few cancer patients. In the future, it is expected that ICB will be a backbone therapy for most patients [318] and therefore it is crucial to improve its efficacy to currently ICB-resistant cancer types. Although this may come in part through the development of novel antagonists of immune checkpoint such as TIM-3, Lag-3, or TIGIT, we believe that this “T-cell centric” approach may provide only limited benefits. Combination approaches may provide better responses; however, in breast cancer, even when associated with neoadjuvant chemotherapy, pembrolizumab, or atezolizumab improved the overall response rate (ORR) of only about 10% compared to chemotherapy as single agent [319,320,321]. It is likely that the reactivation the CD8 T cells in ICB-resistant solid cancers is only partial due to strong local immunosuppression maintained in the TME by high infiltration of macrophages. For instance, breast cancer patients with low infiltration of CD163^+^ macrophages achieved a significantly higher rate of pathologic complete response (pCR) to neoadjuvant chemotherapy [322]. Therefore, we believe that the next breakthrough in cancer treatment may come from triple therapy that will combine ICB and chemotherapy with strategies targeting macrophages. However, and as described in this review, various treatment methods target TAMs and the selection of the best “partner” therapy will require further investigation. Nevertheless, some strategies appear more promising than others. For instance, although attractive and efficient in certain circumstances when associated to chemotherapy [23,50], the strategy of macrophage depletion has several major drawbacks. There is no way to exclusively target the specific macrophage subsets with protumor activities, nor specifically those located in the TME. Thus, the depletion of all macrophages from all tissues has systemic consequences that prevents the prolonged treatment period that is required to sustain macrophage depletion. Peripheral ablation of macrophages is also associated with increased production of monocytes and neutrophils from the bone marrow and treatment cessation is usually associated with a rebound of macrophages in the TME [166]. Also targeting unique immunosuppressive effects has prevented clinical translation because it fails to limit the cause of these TAM subsets or their other protumoral functions. In our opinion, a more promising approach will be the conversion of TAMs into potent antitumor effectors. In this context, intense investigation is focusing on TLR/STING as well as CD40 agonists, and many have already shown safety and tolerability as single agents, allowing now for testing their synergistic effects with ICB and chemotherapy (Table S1).

In addition, TAMs are key players of several immunotherapies. For instance, the effect of anti-CTLA-4 is dependent on the presence of FcR-expressing TAMs for the elimination of Treg cells in the TME [55], and the combination PD-1/CTLA-4 relies on the production of CXCL9 by macrophages [130]. Remarkably, it was shown that macrophages actively participate in tumor clearance in B cell lymphoma treated with rituximab (anti-CD20 mAb) [115] and HER2+ breast cancer treated with trastuzumab and/or pertuzumab [56]. As discussed above, it is plausible that CD47 blockers in combination with anti-HER2 trastuzumab (both targeting the tumor cells) will promote ADCC even for patients whose tumors have become resistant and progressed after trastuzumab [323]. The combination ALX148 + trastuzumab + ramucirumab (VEGFR2) + paclitaxel is being tested in a clinical trial (NCT03013218). Thus, depending on the treatment option combined with macrophage-based immunotherapy, the re-education strategy would be more effective given that macrophages could actively participate in the therapy and induce an efficient antitumor response in the TME. This is in line with the mathematical modeling of T cell–macrophage interactions which determined that macrophage reprogramming into the antitumor subset is the most effective strategy (over depletion or inhibition) [324].

Finally, macrophages display a unique aptitude to penetrate deeply into the core of the tumor, even into hypoxic/necrotic zones, to where antitumor immune cells hardly migrate and cancer therapies are scarcely delivered. This led to the idea of exploiting the macrophage property of intra-tumor invasion by using them as "Trojan Horses" to deliver cytotoxic or stimulatory therapies targeting malignant cells or immune cells, respectively. Using the phagocytic capacity of macrophage to uptake loaded nanoparticles, as a way to deliver therapeutic agents [325] or cytokines [268], was already shown to be successful in mouse models to overcome dense fibroblastic and stroma-rich structures that hinder therapeutic delivery to the tumor. Furthermore, although the usage of particles to activate macrophages antitumor properties has been explored for about 40 years [326] without reaching clinical practice yet, we believe in the potential of this strategy to become a therapeutic reality for cancer patients. The development of nanoparticles made of biomaterial tailored to accurately target and re-educate TAMs may have a lot of potential. In particular, nanoparticles loaded with TLR agonists targeting TAMs are inducing antitumor responses in preclinical models [306].

With all the above, we hope that this review makes the case for turning more attention to developing novel strategies towards programming TAMs as they have clearly emerged as key cancer regulators and potential next-generation immunotherapy targets.

## Supplementary Materials

The following is available online at https://www.mdpi.com/article/10.3390/cells10092364/s1, Table S1: Examples of macrophage-targeting drugs currently investigated in cancer-associated clinical trials.
