# Supplementary material for: Bringing Macrophages to the Frontline against Cancer: Current Immunotherapies Targeting Macrophages
Source: Cells. 2021 Sep 9;10(9):2364. doi: 10.3390/cells10092364 (PMC8464913; doi:10.3390/cells10092364)
Supplement: Supplementary file 1 [file cells-10-02364-s001.zip › cells-1299695-supplementary.pdf]

Target / Drug Name	Company	Identifier	Tumor-type	Therapy Partner	Phase
Macrophage depleting agents					
CSF1					
MCS110 (antagonist mAb)	Novartis	NCT03455764	Melanoma	Dabrafenib, Trametinib	1, 2
CSF1R					
LY3022855 (antagonist mAb)	Eli Lilly	NCT03101254	Melanoma	Vemurafenib, Cobimetinib	1, 2
IMC-CS4 (antagonist mAb)	Eli Lilly	NCT03153410	Pancreatic Adenocarcinoma	Cyclophosphamide Pembrolizumab, GVAX	1
SNDX-6352 (antagonist mAb) Syndax	Pharmaceuticals	NCT03238027	Solid Tumors	Durvalumab	1
Cabiralizumab (antagonist mAb)	Apexigen, Bristol-Myers Squibb	NCT03502330	NSCLC, RCC	APX005M(agonist anti-CD40 mAb) , Nivolumab	1
BLZ945 (CSF-1R inhibitor)	Novartis Pharmaceuticals	NCT02829723	Advanced Solid Tumors	PDR001 (antagonist anti-PD1 mAb)	1, 2
DCC-3014 (CSF1R Inhibitor)	Deciphera Pharmaceuticals LLC	NCT03069469	Advanced Tumors		1, 2
TPX-0022 (MET/CSF1R/SRC Inhibitor)	Turning Point Therapeutics, Inc	NCT03993873	Advanced Solid Tumors		1
Q702 (Axl/Mer/CSF1R Inhibitor)	Qurient Co., Ltd.	NCT04648254	Advanced Solid Tumors		1
PLX3397 (CSF-1R/KIT/FLT3 inhibitor)	Gulam Manji	NCT02584647	Unresectable Sarcoma and Malignant Peripheral Nerve Sheath Tumors	Sirolimus (rapamycin)	1, 2
NMS-03592088 (FLT3/KIT/CSF1R inhibitor)	Nerviano Medical Sciences	NCT03922100	AML, CMML		1, 2
Nilotinib (ABL, KIT, PDGFR/ CSF1R)	National Cancer Institute, France	NCT02029001	Malignant Solid Neoplasms	Everolimus, Sorafenib, Lapatinib, Durvalumab + Tremelimumab	2
Regorafenib (VEGFR/TIE2/KIT/ RET/RAF-1/BRAF/ BRAFV600E/PDGFR/ FGFR/CSF1R)	Apices Soluciones S.L.	NCT04170556	HCC	Nivolumab	1, 2
Inhibitors of recruitment of macrophages to the tumor					
CCR2					
CCX872 (CCR2-inhibitor)	ChemoCentryx	NCT02345408	Pancreatic Adenocarcinoma		1
BMS-813160 (CCR2/5-inhibitor)	Bristol-Myers Squibb	NCT04123379	NSCLC, HCC	Nivolumab	2
CCR5					
Leronlimab (Antagonist anti-CCR5 mAb)	CytoDyn, Inc.	NCT04504942	CCR5+ Locally Advanced or Metastatic Solid Tumors		2
BMS-813160 (CCR2/5-inhibitor)	Bristol-Myers Squibb	NCT03767582	PDAC	Radiation, Nivolumab, GVAX	1, 2
Maraviroc (CCR5-inhibitor)	Pfizer	NCT04721301	Colorectal or Pancreatic Cancer Metastatic	Nivolumab, Ipilimumab	1
Inhibitors of tumor-promoting properties of macrophages					
IDO					

BMS-986205 (IDO1 Inhibitor)	Bristol-Myers Squibb	NCT03854032	Stage II-IV HNSCC	Nivolumab	2
Indoximod (IDO Inhibitor)	NewLink Genetics Corporation	NCT02460367	NSCLC	Docetaxel, Tergenpumatucel-L (vaccine product)	1
Epacadostat (IDO1 Inhibitor)	Incyte Corporation	NCT02785250	Recurrent Ovarian Cancer	DPX-Survivac (vaccine product), Cyclophosphamide	1, 2
KHK2455 (IDO Inhibitor)	Kyowa Kirin, Inc	NCT03915405	Advanced Bladder Cancer	Avelumab (antagonist anti-PDL-1 mAb)	1
Arginase1					
INCB001158 (Arginase inhibitor)	Incyte Corporation	NCT03314935	Solid Tumors	FOLFOX or paclitaxel or gemcitabine/ cisplatin	1
INCB001158 (Arginase inhibitor)	Incyte Corporation	NCT02903914	Advanced/Metastatic Solid Tumors	Pembrolizumab	1,2
Arginase-1 Peptide Vaccine	Herlev Hospital	NCT03689192	Metastatic Solid Tumors		1
Tyro3, Axl and MerTK					
ONO-7475 (AXL inhibitor)	Ono Pharmaceutical Co. Ltd	NCT03176277	Acute Leukemias	Venetoclax (BCL-2 inhibitor)	2
MRX-2843 (Flt3/MerTK Inhibitor)	Meryx, Inc	NCT03510104	Metastatic Solid Tumors	(EGFR inhibitor)	
PF-07265807 (TAM-kinase inhibitor)	Pfizer	NCT04458259	Metastatic Solid Tumors		1
PI3K					
BKM120 (a selective of p110α/β/δ/γ)	National Cancer Institute (NCI)	NCT01816984	Recurrent or Metastatic Head and Neck Cancer	Cetuximab (antagonist EGFR mAb)	2
BYL719	Vanderbilt-Ingram Cancer Center	NCT01791478	HR+ BC	Letrozole (non-steroidal type II aromatase inhibitor)	1
Taselisib / Pictilisib (PI3K inhibitors)	Pfizer	NCT02389842	Advanced Breast Cancer	Palbociclib (CDK4/6 inhibitor)	1
MEN1611 (PI3K inhibitors)	Menarini Group	NCT03767335	Advanced or Metastatic Breast Cancer	Trastuzumab (+/- Fulvestrant)	1
Copanlisib (PI3K inhibitors)	National Cancer Institute (NCI)	NCT03502733	Advanced Cancer and Lymphoma	Ipilimumab, Nivolumab	1
CD47					
HX009 (Anti-CD47 / PD-1 Bifunctional Ab)	Waterstone Hanxbio Pty Ltd	NCT04886271	Advanced Solid Tumors		2
IBI188 (antagonist anti-CD47 mAb)	Innovent Biologics (Suzhou) Co. Ltd.	NCT03717103	Advanced Malignancies		1
TTI-621 (SIRPα / CD47 blocker)	Trillium Therapeutics Inc.	NCT02663518	Hematologic Malignancies Solid Tumors	Rituximab or Nivolumab	1
CC-90002 (antagonist anti-CD47 mAb)	Celgene	NCT02367196	Advanced Solid and Hematologic Cancers	Rituximab	1
Magrolimab (antagonist anti-CD47 mAb)	Gilead Sciences	NCT03248479	Hematological Malignancies, R/R AML, MDS	Azacitidine (Hypomethylating agent)	1
AO-176 (antagonist anti-CD47 mAb)	Arch Oncology	NCT03834948	Solid Tumors	Paclitaxel, Pembrolizumab	1, 2
ALX148 (SIRPα-Fc/ CD47 blocker)	ALX Oncology Inc.	NCT04675333	Advanced HNSCC	Pembrolizumab, Cisplatin/ Carboplatin; 5FU	2
ALX148 (SIRPα-Fc/ CD47 blocker)	ALX Oncology Inc.	NCT04755244	AML	Venetoclax (BCL-2 inhibitor), Azacitidine (Hypomethylating agent)	1, 2

ALX148 (SIRPα-Fc/ CD47 blocker)	ALX Oncology Inc.	NCT03013218	Advanced Solid Tumors and Lymphoma	Pembrolizumab, Trastuzumab or Rituximab, Ramucirumab, Paclitaxel, 5-FU + Cisplatin	1
TG-1801	TG Therapeutics, Inc.	NCT04806035	B-Cell Lymphoma, CLL	Ublituximab (glycoengineered anti-CD20 mAb)	1
CD24					
CD24Fc	Tianhong Li	NCT04552704	Advanced Malignant Solid Neoplasm		1, 2
SIRPa					
CC-95251 (antagonist anti-SIRPa mAb)	Celgene	NCT03783403	Advanced Solid and Hematologic Cancers	Rituximab, Cetuximab	1
BI 765063 (antagonist anti-SIRPa mAb)	OSE Immunotherapeutics	NCT03990233	Advanced Solid Tumors	BI 754091 (PD-1 inhibitor)	1
GS-0189 (antagonist anti-SIRPa mAb)	Gilead Sciences	NCT04502706	R/R NHL	Rituximab	1
Prostaglandin E2 (PGE2) inhibitors					
Etodolac (prostaglandins inhibitor)	Assaf-Harofeh Medical Center	NCT03838029	Pancreatic Neoplasms	Propranolol (beta-adrenergic blocker)	1
Aspirin	Alliance for Clinical Trials in Oncology	NCT02927249	Node Positive HER2- Stage II-III BC		3
Aspirin	University of Virginia	NCT04038489	ER+, HER2- Stage I-III BC	Tamoxifen, Doxorubicin, Cyclophosphamide, Paclitaxel	2
Aspirin	EORTC	NCT02659384	Recurrent Platinum Resistant Ovarian Cancer	Bevacizumab, atezolizumab	2
Aspirin	Sun Yat-sen University	NCT03638297	MSI-H/dMMR or High TMB CRC	BAT1306 (anti-PD-1)	2
Aspirin	Medical University of South Carolina	NCT03245489	Recurrent or Metastatic Head and Neck Cancer	Pembrolizumab Clopidogrel	1
Aspirin	University of California, San Francisco	NCT03396952	Unresectable melanoma	Ipilimumab Pembrolizumab	2
Drugs stimulating macrophage antitumor functions					
TLR3					
Poly ICLC (TLR3 Agonist)	MedImmune LLC	NCT02643303	Advanced, Measurable, Biopsy-accessible Cancers	Durvalumab, Tremelimumab	1, 2
Poly ICLC (TLR3 Agonist)	Oncovir, Inc.	NCT04930783	Metastatic Melanoma	CDX-301 (FLT3 ligand), NEOVAX (personalized neoantigen vaccine), Nivolumab	1
Poly ICLC (TLR3 Agonist)	Celldex Therapeutics	NCT03358719	Myelodysplastic Syndrome or Acute Myeloid Leukemia	DEC-205/NY-ESO-1 Fusion Protein CDX-1401, Decitabine,, Nivolumab	1
Hiltonol Poly-ICLC (TLR3 Agonist)	Gliknik Inc.	NCT02873819	Recurrence of Squamous Cell Carcinoma of the Oral Cavity	GL-0817 (vaccine engineered peptides MAGE-A3) Sargramostim (recombinant version of GM-CSF), cyclophosphamide	2

Hiltonol Poly-ICLC (TLR3 Agonist)	Genocea Biosciences, Inc	NCT03633110	Cutaneous Melanoma NSCLC, SCHN, Urothelial Carcinoma, RCC	GEN-009 personalised neoantigen adjuvanted Vaccine, Nivolumab, Pembrolizumab	1, 2
Hiltonol Poly-ICLC (TLR3 Agonist)	Merck Sharp & Dohme Corp.	NCT03362060	HLA-A2+ Metastatic Triple Negative Breast Cancer	PVX-410 vaccine composed of 4 9-amino acid peptides, Pembrolizumab	1
Poly ICLC (TLR3 Agonist)	Jonsson Comprehensive Cancer Cente	NCT01204684	Brain Tumors	Tumor-lysate pulsed DC vaccination,	2
Poly ICLC (TLR3 Agonist)	National Cancer Institute (NCI)	NCT03206047	Recurrent Ovarian, Fallopian Tube, or Primary Peritoneal Cancer	DEC-205/NY-ESO-1 Fusion Protein CDX-1401, Atezolizumab, Guadecitabine	2
Hiltonol Poly-ICLC (TLR3 Agonist)	Sidney Kimmel Comprehensive Cancer Center at Johns Hopkins	NCT04799431	Pancreatic Cancer Metastatic Colorectal Cancer Metastatic	Neoantigen Vaccine, Retifanlimab (agonist anti-PD1)	
TLR4					
GSK1795091 (TLR4 Agonist)	GlaxoSmithKline	NCT03447314	Advanced Solid Tumors	GSK3174998 (agonist anti-OX40 mAb), GSK3359609 (agonist anti-ICOS mAb), Pembrolizumab	1
TLR7/8					
TransCon (TLR7/8 Agonist)	Ascendis Pharma Oncology Division A/S	NCT04799054	Advanced or Metastatic Solid Tumors	Pembrolizumab	1, 2
BDC-1001 (TLR7/8 Agonist)	Bolt Biotherapeutics, Inc.	NCT04278144	Advanced HER2-Expressing Solid Tumors	Pembrolizumab	1, 2
BDB018 (TLR7/8 Agonist)	Seven and Eight Biopharmaceuticals Inc	NCT04840394	Solid Tumors	Pembrolizumab	1
NKTR-262 (TLR7/8 Agonist)	Nektar Therapeutics	NCT03435640	Locally Advanced or Metastatic Solid Tumor Malignancies	Bempegaldesleukin (PEGylated interleukin-2), nivolumab	1, 2
TLR9					
Tilsotolimod, (TLR-9 Agonist)	Gustave Roussy, Cancer Campus, Grand Paris	NCT04270864	Advanced cancers	Intratumoral Ipilimumab, Intravenous Nivolumab	1
SD-101 (TLR-9 Agonist)	Ronald Levy	NCT03831295	Advanced or Metastatic Solid Malignancies	BMS 986178 (agonist anti-OX40 mAb)	1
SD-101 (TLR-9 Agonist)	Dynavax Technologies Corporation	NCT04050085	Chemotherapy-Refractory Metastatic Pancreatic Cancer	Nivolumab, Radiation Therapy	1
SD-101 (TLR-9 Agonist)	Dynavax Technologies Corporation	NCT03007732	Hormone-Naïve Oligometastatic Prostate Cancer	Pembrolizumab, Leuprolide acetate, Abiraterone Acetate, Prednisone, Stereotactic Body Radiation Therapy	2
CMP-001 (TLR-9 Agonist)	Pfizer	NCT02554812	Advanced Cancer	Avelumab, Utomilumab, PF-04518600, PD 0360324	2
CMP-001 (TLR-9 Agonist)	Pfizer	NCT04401995	Advanced Cancer	Nivolumab	2
DUK-CPG-001 (TLR-9	Agilent Technologies,	NCT02452697	Myeloid or Lymphoid	NK cell-enriched	2

Agonist)	Inc.		Malignancies	donor lymphocyte infusions	
STING/RIG					
E7766 (small STING activator)	Eisai Inc.	NCT04144140	Advanced Solid Tumors or Lymphomas		1
GSK3745417 (small STING activator)	GlaxoSmithKline	NCT03843359	Advanced Solid Tumors	Pembrolizumab	1
SNX281 (small STING activator)	Stingthera, Inc.	NCT04609579	Advanced Solid Tumor Advanced Lymphoma	Pembrolizumab	1
TAK-676 (small STING activator)	Takeda	NCT04420884	Solid Neoplasms	Pembrolizumab	1
TNF-α					
TILT-123 (TNF-a + IL-2 coding oncolytic adenovirus)	TILT Biotherapeutics Ltd.	NCT04217473	Advanced Melanoma	ACT with TILs	1
CD40					
JNJ-64457107	Janssen Research & Development, LLC	NCT02829099	Advanced Stage Solid Tumors		1
2141 V-11 (agonist mAb)	Rockefeller University	NCT04059588	Solid Tumor Cancer of Skin		1
2141 V-11 (agonist mAb)	Rockefeller University	NCT04547777	recurrent malignant glioma	D2C7-IT (dual-specific antibody fragment targeting EGFRwt and EGFRvIII and recombinant Pseudomonas exotoxin)	1
LVGN7409 (Agonist mAb)	Lyvgen Biopharma Holdings Limited	NCT04635995	Advanced or Metastatic Malignancy		1
SL-172154 (SIRPα-Fc-CD40L)	Shattuck Labs, Inc.	NCT04406623	Ovarian Cancer		1
APX005M (agonist mAb)	Apexigen, Inc.	NCT03502330	NSCLC, RCC	Cabiralizumab (CSF1R mAb), Nivolumab	1
APX005M (agonist mAb)	Apexigen, Inc.	NCT04130854	Locally Advanced Rectal Adenocarcinoma	mFOLFOX, and Radiation Therapy 5Gy x 5 days	2
APX005M (agonist mAb)	Apexigen, Inc.	NCT03165994	Esophageal and GastroEsophageal Cancer	Radiation Therapy, Paclitaxel, Carboplatin and surgical resection of tumor	2
CDX-1140 (agonist mAb)	Celldex Therapeutics	NCT04491084	Lung Cancer	CDX-301 (FLT3 ligand), Stereotactic Radiotherapy	1 ,2
CDX-1140 (agonist mAb)	Celldex Therapeutics	NCT03329950	Lung Cancer	FLT3 Ligand (CDX-301), Pembrolizumab, Chemotherapy	1 ,2
CDX-1140 (agonist mAb)	Celldex Therapeutics	NCT04364230	Melanoma	6MHP, NeoAg-mBRAF, PolyICLC	1, 2
SEA-CD40 (agonist mAb)	Seagen Inc.	NCT02376699	Solid tumors	SEA-CD40 (IV) + pembrolizumab + gemcitabine + nab-paclitaxel	1
RO7300490, A Fibroblast Activation Protein-α (FAP) Targeted CD40 Agonist,	Hoffmann-La Roche	NCT04857138	Solid tumors	Atezolizumab	1
Autologous Tumor Cell-Based Vaccine	H. Lee Moffitt Cancer Center and Research	NCT00101101	Mantle Cell Lymphoma	Cyclophosphamide, Doxorubicin,	1

producing GM-CSF and CD40L	Institute			Vincristine, Prednisone, Dexamethasone, IL-2	
CMN-001 autologous, tumor antigen-loaded dendritic cell immunotherapy, electroporated with CD40L RNA	CoImmune	NCT04203901	Advanced Renal Cell Carcinoma	Nivolumab, Ipilimumab, Lenvatinib, Everolimus	2
NG-350A (Oncolytic Adenoviral Vector Which Expresses an Anti-CD40 mAb)	PsiOxus Therapeutics Ltd	NCT03852511	Metastatic Cancer Epithelial Tumor		1
LOAd703 (Oncolytic adenovirus which expresses the transgenes trimerized membrane-bound isoleucine zipper (TMZ) TMZ-CD40L and 41BBL)	Lokon Pharma AB	NCT04123470	Malignant Melanoma	Atezolizumab	1, 2
CAR-Macrophages					
CT-0508 (anti-HER2 Chimeric Antigen)	Carisma Therapeutics Inc	NCT04660929	HER2 Overexpressing Solid Tumors		1

Table S1: Examples of macrophage-targeting drugs currently investigated in cancer-associated clinical trials (only including active and/or recruiting, interventional trials from http://clinicaltrials.gov/ until 01 August 2021). Of note, we selected examples of trials prioritising combinatorial therapies over the individual therapy. The list is organised following depletion, inhibition of recruitment or protumor functions, activating agents.

Abbreviations: Acute Myeloid Leukemia: AML; Chronic Lymphocytic Leukemia: CLL; Chronic Myelomonocytic Leukemia: CMML; Colorectal Cancer: CRC; Hepatocellular Carcinoma: HCC; Head and Neck Squamous Cell Carcinoma: HNSCC; Hormone Receptor-Positive Metastatic Breast Cancer: HR+ BC; Myelodysplastic Syndrome: MDS; Non-Hodgkin Lymphoma: NHL; Non-Small Cell Lung Cancer: NSCLC; Pancreatic Ductal Adenocarcinoma: PDAC; Renal Cell Carcinoma: RCC; Relapsed/Refractory: R/R.
Adoptive Cell Therapy: ACT; Inducible T-cell co-stimulatory: ICOS; Platelet-derived growth factor receptors: PDGFR; Tumor Infiltrating Lymphocytes: TILs.
